# Supplementary material for: Circulating Interleukins as Biomarkers in Non-Small Cell Lung Cancer Patients: A Pilot Study Compared to Normal Individuals
Source: Diseases. 2024 Sep 18;12(9):221. doi: 10.3390/diseases12090221 (PMC11430979; doi:10.3390/diseases12090221)
Supplement: Supplementary file 1 [file diseases-12-00221-s001.zip › diseases-3204745-supplementary.pdf]

Supplemental Table S1. Published lung cancer studies assessing IL-11 levels in various biological sources.

Reference	Pathology	Comparison	Source / Matrix	Collection method	Concentration Method	Assay Method	Assay kit / Manufacturer	Assay Range (pg/ml)	Findings (pg/ml)	Remarks / Coimments
PMID: 27524264	Lung Cancer (LC) with or without chronic obstructive pulmonary disease (COPD)	1st validation cohort: Controls (n=20) vs. COPD (n=29) vs. LC (n=50) vs. COPD with LC (n=50)	Bronchoalveolar lavage fluid (BALF)	BALF samples were obtained by intillation of 40-60 ml of 0.9% sterile saline, sterile filtered, centrifuged to obtain supernatant stored in 2 ml aliquots, and frozen until usage.	Vacuum concentrated for proteomic studies: 4-8 ml of BALF sample per patient was vaccum concentrated to 1.5-2 ml over a 2-6 hour period. Protein concentration was assessed by RCDC method	ELISA	DY218 (R&D Systems)	31.3 – 2,000 pg/ml	**1st validation cohort:**<br>1) LUAD: median 107 pg/mL, IQR = 61–196<br>2) LUAD w/ COPD: median 72 pg/mL, IQR = 51–137<br>3) COPD alone, SCC or SCC w/ COPD did not significantly differ to controls<br>4) Controls: unspecified concentration.<br>5) Optimal diagnostic cut-off = 42.0 pg/mL; % Sensitivity = 78.8%, % Specificity = 88.7% in patients with adenocarcinoma	1) Unclear how the controls were selected to undergo BALF collection.<br>2) Unclear if reported concentrations have been corrected for concentration factor if concentrated samples were used to run ELISA.<br>3) Unclear on fold-dilutions (if any) for assay.<br>4) Unclear why optimal diagnostic cut-off for diagnosis of adenocarcinoma (ROC curve) was reduced in 2nd validation cohort from 42 to 29.5 pg/mL.<br>5) Graphical presentation of IL-11 concentration by ELISA in controls depict 0 values below the assay detection range.
		2nd validation cohort: Controls (n=20) vs. LC (n=66) vs. COPD with LC (n=74)							**2nd validation cohort:**<br>1) LUAD: median 104 (IQR: 53–166);<br>2) controls: unspecified concentration.<br>3) Diagnostic cut-off = 29.5 pg/mL; % Sensitivity = 90.6%, % Specificity = 83% in patients with adenocarcinoma	
PMID: 15072171	Pleural effusions from tuberculosis (TB), bronchial carcinomas (BC), secondary carcinomas (CA), congestive heart failure (CHF), and pneumonias (PN)	Pleural effusion and plasma of TB (n=16), BC (n=28), CA (n=17), CHF (n=9), PN (n=6)	Pleural effusion	The pleural fluid was extracted into EDTA-containing tubes. Aliquotted and stored at −70°C for later analysis.	No attempts on any sample concentration was mentioned.	ELISA	D1100 (R&D Systems)	31.3 – 2,000 pg/ml	1) "Only 36 of total 76 patients had positive IL-11 values in the pleural effusion" comprising of 21 patients in TB, CA groups (high) vs. 15 patients in BC, PN groups (low).<br>2) IL-6 levels were found in effusions of BC patients (8243±7722 pg/ml) with min whiskers in box-and-whiskers plots indicating 0 or close to zero values.<br>3) IL-11 levels were found in effusions in various disorders with min whiskers in box-and-whiskers plots indicated at zero values.<br>4) At diagnostic cut-off value of 206 pg/ml, TB and CA patients were assigned to high IL-11 group, whereas BC and PN patients were designated low IL-11 group. No sensitivity could be calculated at 95% specificity. Only at 87% specificity, a sensitivity of 38% could be found. Due to the fact that the ROC curve runs near the diagonal, no diagnostic separation was possible.	1) No compatible control pleural effusion/plasma from healthy controls.<br>2) No serum from diseased subjects with pleural effusions.<br>3) No mention of fasting status at time of blood collection.<br>4) Unclear on fold-dilutions (if any) for assay.<br>5) Mixed BC subjects comprising of lung adenocarcinoma (n=12), undeterminated type NSCLC (n=5), small cell carcinoma (n=4), squamous cell carcinoma (n=3) and undifferentiated type (n=4).<br>6) 15 out of 28 BC pleura effusion were detected within the dynamic detection range. None of the paired plasma in same subjects recorded detectable IL-11 levels.<br>7) Graphical presentation of IL-11 concentration by ELISA in groups depict 0 values (min value of box-and-whiskers plot) below the assay detection range.
			Plasma	Blood was withdrawn from peripheral vein and collected in tubes containing EDTA. Aliquotted and stored at −70°C for later analysis.					1) "IL-11 could be found only in pleural effusions, similar to IL-6" i.e. IL-6 and IL-11 levels in peripheral blood (either plasma or serum) were under the detection limit of the current kit assay	
		Control sera (n=6)	Serum	Blood was withdrawn from peripheral vein and collected in plain tubes. Aliquotted and stored at −70 °C for later analysis.						
PMID: 31562055	Non-small cell lung cancer (NSCLC)	Adenocarcinoma (n=5) vs. squamous NSCLC (n=22)	Plasma	Serial collection of peripheral blood for plasma before starting treatment (T0), since beginning nivolumab monotherapy (T1), and progression of disease (2), if applicable.<br>Collection tube for plasma is undetermined.	No attempts on any sample concentration was mentioned.	ELISA	D1100 (R&D Systems)	31.3 – 2,000 pg/ml	1) T0 (baseline prior to Nivolumab treatment):<br>Adenocarcinoma: mean 12.24 ± 6.01 (SD)<br>Squamous: 10.60 ± 6.04 (SD)<br>2) No significant differences in IL-11 levels were found between nibolumab responders vs. non-responders at all time points T0, T1, and T2.<br>3) Non-significant decrease in IL-11 levels were observed at T1 (vs. T0).	1) IL-11 concentrations reported are below dynamic detection range specified by assay.<br>2) Unclear if concentration of samples were performed for assay.<br>3) Unclear on fold-dilutions (if any) for assay.<br>4) No mention of fasting status at time of blood collection.
PMID: 34142593	NSCLC	NSCLC (n=91) vs. healthy volunteers (n=72)	Serum	5 ml of fasting venous blood collected in coagulation tube, centrifugation for serum collection, stored at −80 °C.<br>No mention about aliquots, and undetermined blood tube.	No attempts on any sample concentration was mentioned.	ELISA	E-EL-H5022 (Elabscience)	31.3 – 2,000 pg/ml	1) Serum IL-11 levels:<br>NSCLC: median 193.80 (IQR: 123.50–323.60)<br>Healthy volunteers: median 44.51 (IQR: 23.24–77.05)	1) IL-11 concentrations in EBCs are generally below dynamic detection range specified by assay.<br>2) Unclear if concentration of samples were performed for assay.<br>3) Unclear on fold-dilutions (if any) for assay.
			Exhaled breath condensates	EBC was collected using EcoScreen condenser (Eric Jaeger Company), temperature set at −20 °C and collection time for 20 mins.<br>Given limitations of subject cooperation only n=64 NSCLC and n=63 healthy volunteers were collected.					2) EBC IL-11 levels:<br>NSCLC: median 45.86 (IQR: 26.73–75.76)<br>Healthy volunteers: median 11.47 (IQR: 8.07–17.21)	
